# Experimental Study on the Expression of IL-1*β* and bFGF in Wound Healing Process of Rabbit Cutaneous Infective Wound in Liu-He-Dan

**DOI:** 10.1155/2017/7230178

**Published:** 2017-12-10

**Authors:** Changyao Lv, Jing Wu, Hongbo He

**Affiliations:** Department of Integrated Traditional Chinese and Western Medicine, West China Hospital of Sichuan University, Chengdu 610041, China

## Abstract

**Objective:**

This study applied Liu-He-Dan (LHD) to treat the infective wounds of rabbits to explore the mechanism of LHD in promoting wound healing.

**Method:**

Five circular infective incisions were generated on the back of each rabbit. Wound dressings were performed every day since postoperative day 1. Ten rabbits were euthanized on days 3, 7, 14, and 21. Each specimen was divided into two parts, one was used for detecting interleukin-1 beta (IL-1*β*), and the other one was used for detecting basic fibroblast growth factor (bFGF).

**Result:**

The content of IL-1*β* in the model group was higher than those in the other groups (*P* < 0.05). The content of IL-1*β* in the treatment group was lower than the other groups on days 14 and 21. The expression of bFGF in treatment group is significant on days 3, 7, and 14, compared with traditional Chinese medicine group and model group. The expression of bFGF has no significant difference with Western group.

**Conclusion:**

The research approved that LHD could specifically suppress the expression of IL-1*β* and upregulate the expression of bFGF in the wound, decreasing the release of inflammatory factor of the infective wounds and promoting the healing of the infective wounds.

## 1. Introduction

The main etiology of anal fistula is the infection of anal glands, which locates in the internal sphincter. Inflammation, trauma, or fecalith causes the obstruction of the drain ducts of the gland; thus, abscess forms in the intersphincteric space, and 30%~40% of the cases may develop into the anal fistula. The mainly therapeutic modality in China is anal fistulectomy. Dressing change has to be performed after defecation every day at least for a month, which is considered an important therapeutic method in promoting wound healing, but this method increases patients' pain and lengthens the time of wound healing; both are problems in clinical treatment. Traditional Chinese medicine (TCM) has been adopted to accelerate postoperative wound healing for thousands of years. Liu-He-Dan (LHD) is a traditional herbal ointment, which has been proved to be effective in treating acute inflammation, such as acute pancreatitis. It will reduce inflammation and relieve pain. However, there were few reports on the research on controlling chronic inflammation reaction and curative effect on postoperative wound. This study applied LHD to treat the infective wounds of rabbits. We performed the biochemical and immunohistochemical evaluation to explore the mechanism of LHD in promoting wound healing. 

## 2. Materials and Methods

### 2.1. Animals and Wounds Model

Forty healthy New Zealand white rabbits were purchased from West China Laboratory Animal Service Station, Sichuan University (Chengdu, China, Scxk (chuan) 2013-14). Forty rabbits were kept separately in clean cages and had equal amounts of standard food and water. They were housed in temperature-controlled (18~24°C)and humidity-controlled (40%–60%) rooms with 12-hour light/dark photoperiods and allowed to adapt to their environment for one week before the experiment.

First, rabbits were anesthetized with 0.7% pentobarbital sodium via auricular veins (6 ml/Kg). Then, their back hair was shaved (12 cm × 10 cm), and the wound sites were disinfected with povidone iodine. Next, five full-thickness circular excisional skin wounds (20 mm in diameter and deep into the fascia) were created on the back of each rabbit with scissors and forceps. Each rabbit had two longitudinal wounds on the left side of the back and three longitudinal wounds on the right side. All the wounds were divided into five groups, control group, model group, treatment group (LDH), Western medicine group (calcium alginate), and traditional Chinese medicine group (shikonin oil), according to the counterclockwise order from the upper left. The wounds were covered with 2 cm diameter circular gauze and infected with 1 ml* Staphylococcus aureus*. The rabbits were kept in cages during the study period, with equal amounts of food and water.

The wounds dressings were changed for the first time on postoperative day 1, during the 21-day experiment period; the rabbits' wounds dressings were carefully changed every day. The calcium alginate and shikonin oil were adopted to the wound surface areas, and LHD was adopted to the surface areas around the wound, with dressing at 24-hour intervals with disposable applicators. Of note, rabbits were excluded from the experiment if dead.

### 2.2. Wound Healing Rate

On days 3, 7, 14, and 21, ten rabbits were selected randomly to be euthanized, and their wounds areas were drawn on transparent film and calculated by putting the transparent film on the electrocardiograph paper. The percentage of wound closure was calculated as follows: wound closure (%) = (area of original wound-area of actual wound)/area of original wound × 100.

### 2.3. Histopathological Evaluation of Wound Healing

Circular full-thickness skin from wounds sites of five groups was taken (the wound site with a margin of 2 mm) after the rabbits were euthanized. Each specimen was divided into two parts; one was immediately fixed in buffered formaldehyde (10% formalin) and then sent for histopathological assessments; the specimens were embedded in paraffin, sectioned into 5 *μ*m slices by microtome (Leica, Germany, RM2155), and stained with hematoxylin and eosin. Histopathological evaluation of physiological parameters including the following criteria indicates the wound healing process: acute and chronic inflammatory infiltrates, granulation tissue, collagen deposition, neovascularization, and reepithelialization. Samples were assessed by microscope (Olympus, Tokyo, Japan, BX5), and images were analyzed by CCD (Olympus, Tokyo, Japan, DP73). Two investigators who assessed tissue samples and analyzed images in this study were blinded to the agents given.

### 2.4. Biochemical Evaluation of Wound Healing

The other part of specimen was irrigated with PBS, and the homogenate was centrifugated at 10,000 rpm at 4°C for 15 min. The content of IL-1*β* in the granulation tissue was determined by Elisa kit (Beijing Biosynthesis Biotechnology Co., Ltd.) according to the manufacturer's instructions.

### 2.5. Immunohistochemical Analysis of Wound Healing

The paraffin embedded specimens were sectioned into 5 *μ*m slices, The expression of bFGF in the granulation tissue was determined by immunocytochemistry and rabbit anti-TNF alpha antibody (Beijing Biosynthesis Biotechnology Co., Ltd.) according to the manufacturer's instructions.

## 3. Statistical Analysis

The results of expression of IL-1*β* were presented as mean ± standard deviation (SD). Statistical comparisons were made with SNK-q (SPSS Statistics software version 17; Chicago, Illinois, USA). The values of *P* < 0.05 were considered statistically significant.

## 4. Results

### 4.1. Wound Healing Rate

**Figure 1:**
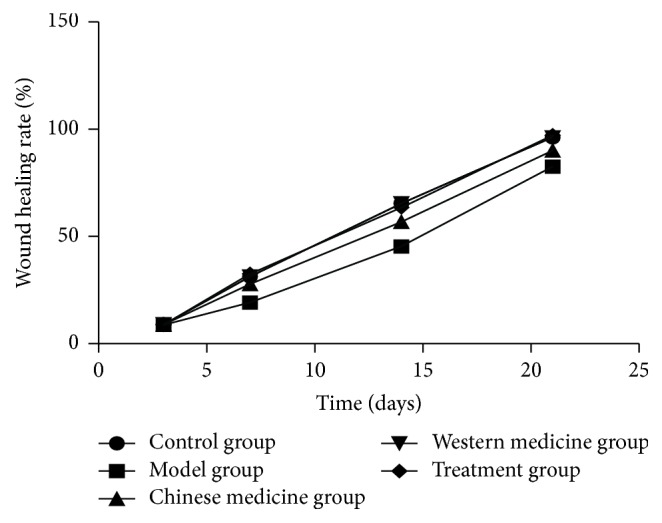
Wound healing rate.

**Figure 2:**
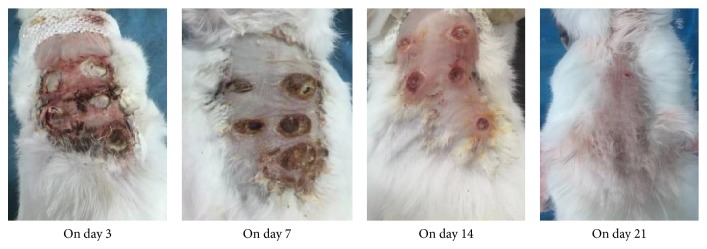
The pictures of wounds healing of days 3, 7, 14, and 21.

All rabbits survived during the whole time of the experiment. The results indicated no statistically significant difference among the five groups on day 3. The wound healing rate of treatment group, Western medicine group, and control group were significantly increased compared with model group and traditional Chinese medicine group on days 7, 14, and 21 (*P* < 0.05); the results indicated no statistically significant difference among the three groups (*P* > 0.05) (Figures 1 and 2).

### 4.2. Histopathological Examinations

**Figure 3:**
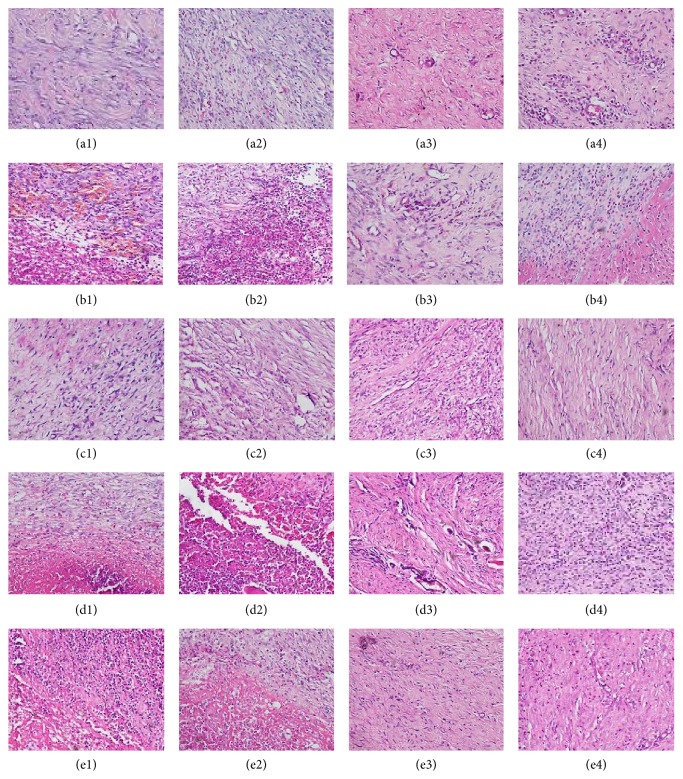
The images of H&E slide of all groups on days 3, 7, 14, and 21. ((a1) control group on day 3, (a2) control group on day 7, (a3) control group on day 14, (a4) control group on day 21, (b1) model group on day 3, (b2) model group on day 7, (b3) model group on day 14, (b4) model group on day 21, (c1) traditional Chinese medicine group on day 3, (c2) traditional Chinese medicine group on day 7, (c3) traditional Chinese medicine group on day 14, (c4) traditional Chinese medicine group on day 21, (d1) treatment group on day 3, (d2) treatment group on day 7, (d3) treatment group on day 14, (d4) treatment group on day 21, (e1) Western medicine group on day 3, (e2) Western medicine group on day 7, (e3) Western medicine group on day 14, and (e4) Western medicine group on day 21).

Swelling, acute inflammation infiltration, necrotic tissue, and mass inflammatory cell were observed in all the groups on day 3. The inflammatory cell and necrotic tissue were significantly reduced in the treatment group, and inflammatory infiltrates and granulation tissue were observed on day 7. On day 14, skin appeared; fibroblasts, granulation tissue, and new blood vessels were observed. On day 21, the complete wounds closures and collagen deposition, vascular maturation, and scar formation were observed in all groups (Figure 3).

### 4.3. Biochemical Examinations

**Figure 4:**
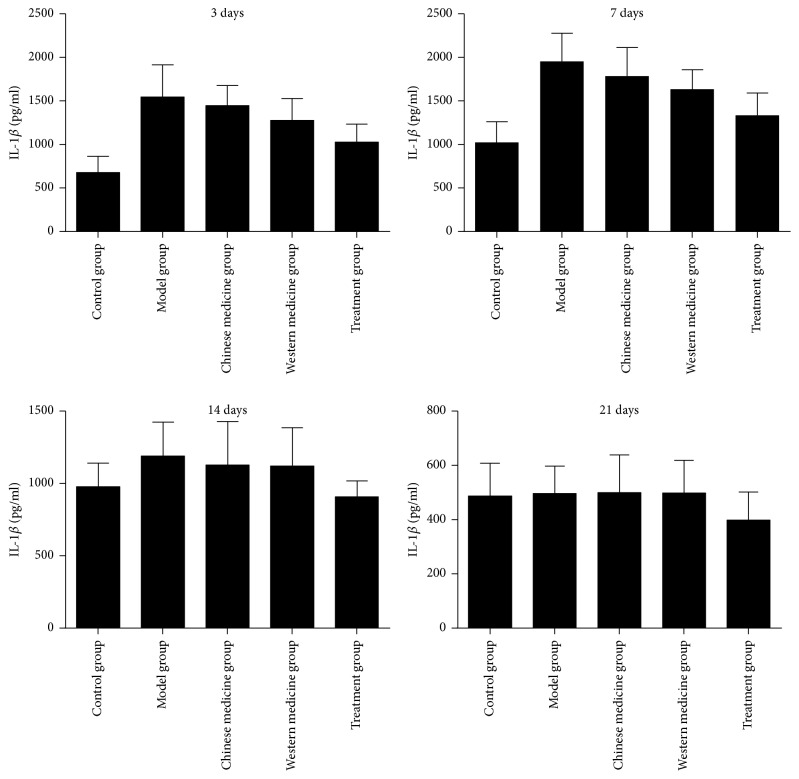
The content of IL-*β* in granulation tissues.

The content of IL-1*β* in model group was higher than in the other groups during the experiment (*P* < 0.05). The content of IL-1*β* in control group was lower than those in the other groups on day 3 and day 7 (*P* < 0.05), but the content of IL-1*β* in the treatment group was lower than the control group on day 14 and day 21 (*P* < 0.05). The content of IL-1*β* in the treatment group was lower than traditional Chinese medicine group, Western medicine group, and model group on days 3, 7, and 21 (*P* < 0.05). The content of IL-1*β* in the Western medicine group was lower than traditional Chinese medicine group on day 3 and day 7 (*P* < 0.05), but there was no significant difference between the two groups on day 14 and day 21 (Figure 4).

### 4.4. Immunohistochemical Analysis

**Figure 5:**
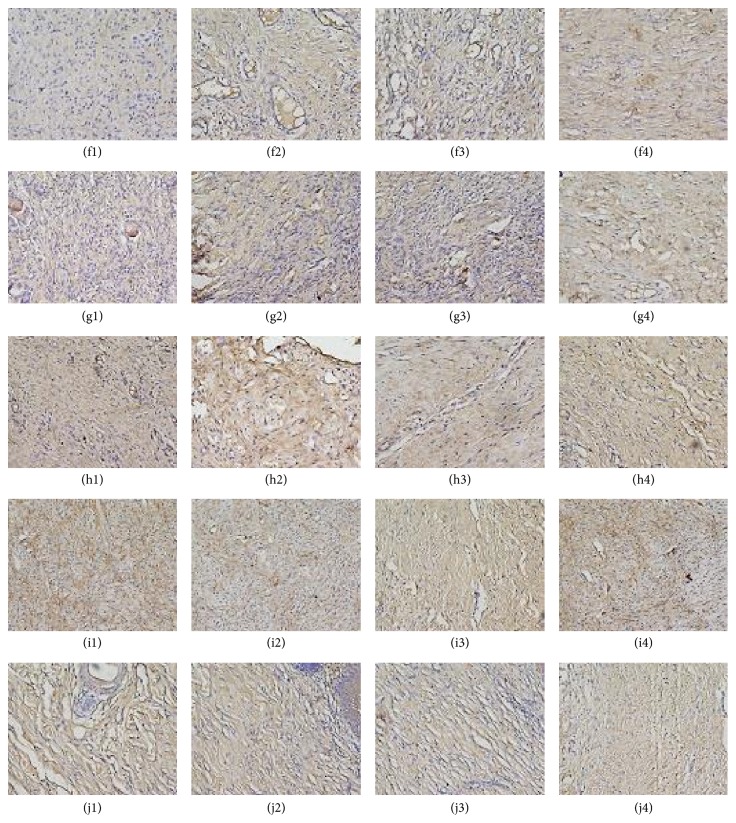
The images of immunohistochemical staining of all groups. ((f1) control group on day 3, (f2) control group on day 7, (f3) control group on day 14, (f4) control group on day 21, (g1) model group on day 3, (g2) model group on day 7, (g3) model group on day 14, (g4) model group on day 21, (h1) traditional Chinese medicine group on day 3, (h2) traditional Chinese medicine group on day 7, (h3) traditional Chinese medicine group on day 14, (h4) traditional Chinese medicine group on day 21, (i1) treatment group on day 3, (i2) treatment group on day 7, (i3) treatment group on day 14, (i4) treatment group on day 21, (j1) Western medicine group on day 3, (j2) Western medicine group on day 7, (j3) Western medicine group on day 14, and (j4) Western medicine group on day 21).

The expression of bFGF in the treatment group has a significant expression on days 3, 7, and 14, compared with the traditional Chinese medicine group and the model group. The expression of bFGF has no significant difference when compared with Western group (Figure 5).

## 5. Discussion

Anal fistula is a common disease in clinical treatment; the main treatment in China is the anal fistulectomy, which is accepted by most clinical doctors. Wounds healing needs at least a month, which costed more, made time extension of the hospital stays, and reduced living quality. Traditional Chinese medicine has been adapted to postoperative wound and accelerating wound healing for thousands years. However, it lacked convincing evidence, and the mechanism is unclear. LHD is a kind of Chinese herbal ointment, consisting of* Rheum officinale* Baill.,* Phellodendron chinense* Schneid.,* Bletilla striata*,* Mentha haplocalyx* Briq.,* Angelica dahurica* Benth., honey, flour, and other herbs. LHD is adapted to acute inflammation, pancreatitis, adhesive ileus, skin boil, sores, phlebitis, superficial infection, and acute gouty arthritis, which has been proved to be effective. In the previous study, LHD could reduce serum proinflammatory cytokines, such as IL-6 and CRP [1, 2]. Evidence on LHD applied to postoperative infective wound is insufficient; this study examined the effects of this product in the process of infective wound healing [3].

Wound healing is a response to an injury, which is complex and dynamic sequence of event. The event requires constant communication among participating tissues. This process comprises four phases: hemostasis, inflammation, proliferation, and remodeling. The process includes vascular injury, fibrin-fibronectin clot formation, platelet recruitment, migration, and proliferation of vascular endothelial cells, fibroblasts, and granulation tissue formation. In the inflammation phase, inflammatory cells migrate into the wound to promote the inflammation phase [4–6]. Inflammatory cells could communicate with each other through cell-cell contact, cytokines, and growth factors [7]. IL-1*β* is a major proinflammatory cytokine produced by a variety of immune cells including keratinocytes, monocytes, and other epithelial cells, involved in the inflammatory response [8, 9]. IL-1*β* upregulates adhesion molecule expression, activates neutrophils, promotes the secretion of other proinflammatory cytokines, and further contributes to local inflammatory response [10], which is thought to be deleterious in the wound repair.

In the proliferation phase, growth factors as bFGF and VEGF are effectors to stimulate tissue deposition and epithelialization, accelerating wound healing. bFGF is a member of a large FGF family and induces angiogenesis, endothelial cell migration, and fibroblast proliferation in the wound healing process [11–14].

Generally, the direct and effective parameters involved in wound healing effects are wound healing rate, wound healing duration, and pathological analysis of wounds [15]. Persistent inflammation in wound generates delay in wound healing and wound chronicity [16]. Our data showed that LHD would inhibit the content of IL-1*β*, IL-1*β* has a significant expression on day 7 in all the groups, and then the content of IL-1*β* was suppressed on days 14 and 21. However, the content of IL-1*β* in treatment group was lower than in the other groups on days 3, 7, 14, and 21. LHD would promote the expression of bFGF, on days 3, 7, and 14; the expression of bFGF in the treatment group was stronger than the traditional Chinese group and the model group; however, there was no significant difference when compared with Western group. Thus, the wound healing rate of treatment group is higher than the other groups on days 7 and 21.

Our result indicates that LHD can inhibit the content of IL-1*β* and upregulate the expression of bFGF. Thereby, LHD alleviates the duration of inflammation and increases neovascularization, vascular endothelial cells, and fibroblasts in the granulation tissue. LHD promotes the expression of growth factors, bFGF, accelerating proliferation of resident fibroblasts; thus the number of fibroblasts increases.

In conclusion, LHD can promote cutaneous excisional wound healing by inhibiting the expression of IL-1*β* and upregulating the expression of bFGF, revealing primary mechanism to accelerate wound healing, but further studies are still needed.
